# Antitumor effect of a small-molecule inhibitor of KRAS^G12D^ in xenograft models of mucinous appendicular neoplasms

**DOI:** 10.1186/s40164-023-00465-4

**Published:** 2023-12-08

**Authors:** Mari C. Vázquez-Borrego, Melissa Granados-Rodríguez, Florina I. Bura, Ana Martínez-López, Blanca Rufián-Andújar, Francisca Valenzuela-Molina, Lidia Rodríguez-Ortiz, Sergio Haro-Yuste, Ana Moreno-Serrano, Rosa Ortega-Salas, Rafael Pineda-Reyes, Carmen Michán, José Alhama, Antonio Romero-Ruiz, Álvaro Arjona-Sánchez

**Affiliations:** 1 Maimonides Biomedical Research Institute of Córdoba, Córdoba, Spain; 2 Department of Biochemistry and Molecular Biology, University of Córdoba, Córdoba, Spain; 3 Surgical Oncology Unit, Surgery Department, Reina Sofía University Hospital, Córdoba, Spain; 4 Pathology Unit, Reina Sofía University Hospital, Córdoba, Spain; 5 Department of Cell Biology, Physiology, and Immunology, University of Córdoba, Córdoba, Spain

**Keywords:** Cancer, Pseudomyxoma Peritonei, Mucin, KRAS, MRTX1133

## Abstract

Pseudomyxoma peritonei (PMP) is a rare disease characterized by a massive accumulation of mucus in the peritoneal cavity. The only effective treatment is aggressive surgery, aimed at removing all visible tumors. However, a high percentage of patients relapse, with subsequent progression and death. Recently, there has been an increase in therapies that target mutated oncogenic proteins. In this sense, *KRAS* has been reported to be highly mutated in PMP, with KRAS^G12D^ being the most common subtype. Here, we tested the efficacy of a small-molecule KRAS^G12D^ inhibitor, MRTX1133, in a high-grade PMP xenograft mouse model carrying a KRAS^G12D^ mutation. The results obtained in this work showed a profound inhibition of tumor growth, which was associated with a reduction in cell proliferation, an increase in apoptosis, and a reduction in the MAPK and PI3K/AKT/mTOR signaling pathways. In conclusion, these results demonstrate the high potency and efficacy of MRTX1133 in KRAS^G12D^-PMP tumors and provide a rationale for clinical trials.

**Supplementary Information:**

The online version contains supplementary material available at 10.1186/s40164-023-00465-4.

## To the editor

Pseudomyxoma peritonei (PMP) is a rare clinical entity characterized by progressive accumulation of mucinous gelatinous material in the peritoneal cavity, without extraperitoneal growth or distant metastases [1]. This disease is categorized into three groups by the Peritoneal Surface Oncology Group International (PSOGI): (i) Low-Grade (LG-PMP); (ii) High-Grade (HG-PMP); and (iii) PMP with the presence of signet ring cells (SRC-PMP) [2]. The most effective treatment option for PMP includes cytoreductive surgery (CRS) associated with hyperthermic intraperitoneal chemotherapy (HIPEC), which aims to remove all visible tumor within the peritoneum [3]. Despite this therapeutic effort, a high percentage of patients will develop relapse with subsequent progression and death due to the absence of effective treatment options [4].

*KRAS* is one of the most frequently mutated oncogenes in various cancer [5] and is reported to be mutated at a median frequency of 78% in PMP [5], with KRAS^G12D^ being the most common subtype [6]. KRAS^G12D^ promotes uncontrolled cell proliferation and survival by constitutively activating KRAS protein, a critical component of the MAPK and PI3K/AKT signaling pathways [7]. MRTX1133 is an investigational small-molecule inhibitor developed by Mirati Therapeutics Inc. (CA, USA). It was designed to selectively bind to the mutant KRAS^G12D^ protein and inhibit its activity [7]. MRTX1133 has proven to be an effective treatment for animal models of KRAS^G12D^-mutated colorectal and pancreatic cancers [8, 9]. Importantly, this treatment has proven to be highly selective for KRAS^G12D^ due to its binding to a specific histidine (H95), which is not conserved in wild-type KRAS, HRAS, or NRAS [10].

In this study, we first tested the direct effect of MRTX1133 on tumor progression in a xenograft mouse model of PMP with the KRAS^G12D^ mutation.

First, we describe and validate a HG-PMP xenograft mouse model with < 50% signet ring cells that exhibited a growth pattern very similar to its human counterpart (see Additional Information: methods). Additionally, immunohistochemical analyses showed that the expression patterns of specific markers, such as MUC2, CK7, CK20, P53, and CDX2, were maintained and consistent in the patient-derived xenograft (PDX) mouse model compared to the original human sample (Table S1). These results are consistent with those generated by Flatmark et al. [11]. Interestingly, we found that our PMP PDX mouse model carried the KRAS^G12D^ mutation (see Additional Information: methods and Table S2), making it a perfect candidate for testing therapies that target this specific mutation, such as MRTX1133.

**Fig. 1:**
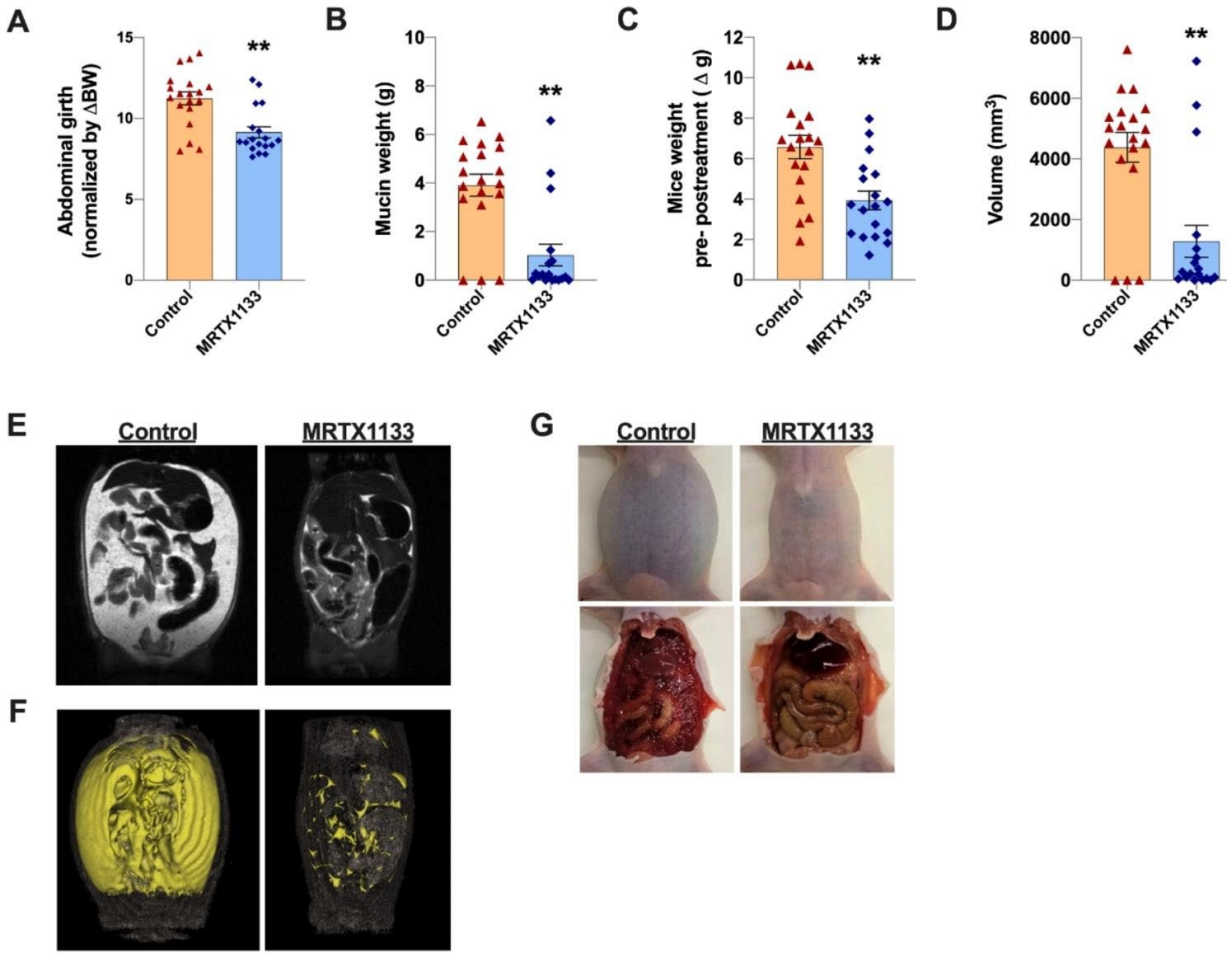
MRTX1133 (30 mg/kg) reduces tumor growth in a HG-PMP xenograft mouse model. **(A–C)** Abdominal girth (normalized by body weight gain), mucinous tumor weight (g) and pre/post-treatment mouse weight gain (calculated as the difference between the pre-treatment mouse weight and the weight before sacrifice) measured after sacrifice in MRTX1133-treated (n = 18) and control mice (n = 19). **(D)** Quantification of an estimated mucinous tumor volume (mm^3^) within mouse peritoneum using MRI T2-weighted images in treated (n = 18) and control mice (n = 19). **(E)** Representative MRI T2-weighted images of MRTX1133-treated and control mice. Mucin appears as hypointense regions in the images. **(F)** Representative 3D images of mouse abdomens showing the distribution of mucin within peritoneumin treated and control mice. Mucin appears highlighted in yellow in the images. **(G)** Representative images of a control (left images) and a MRTX1133-treated mouse (right images) at sacrifice. Mucin appears as a viscous liquid throughout the peritoneum in control mice. Data are represented as the mean ± SEM. ** p < 0.001.

MRTX1133-treated HG-PMP PDX mice showed profound tumor growth inhibition based on the reduction in abdominal girth, mucin weight, mucin volume, and pre/post-treatment weight gain compared to the control group (Fig. 1). These results are consistent with the reduction in cell viability observed in vitro in several cancer cell lines and tumor regression observed in vivo in mouse models of pancreatic and colorectal cancer [7, 9].

**Fig. 2:**
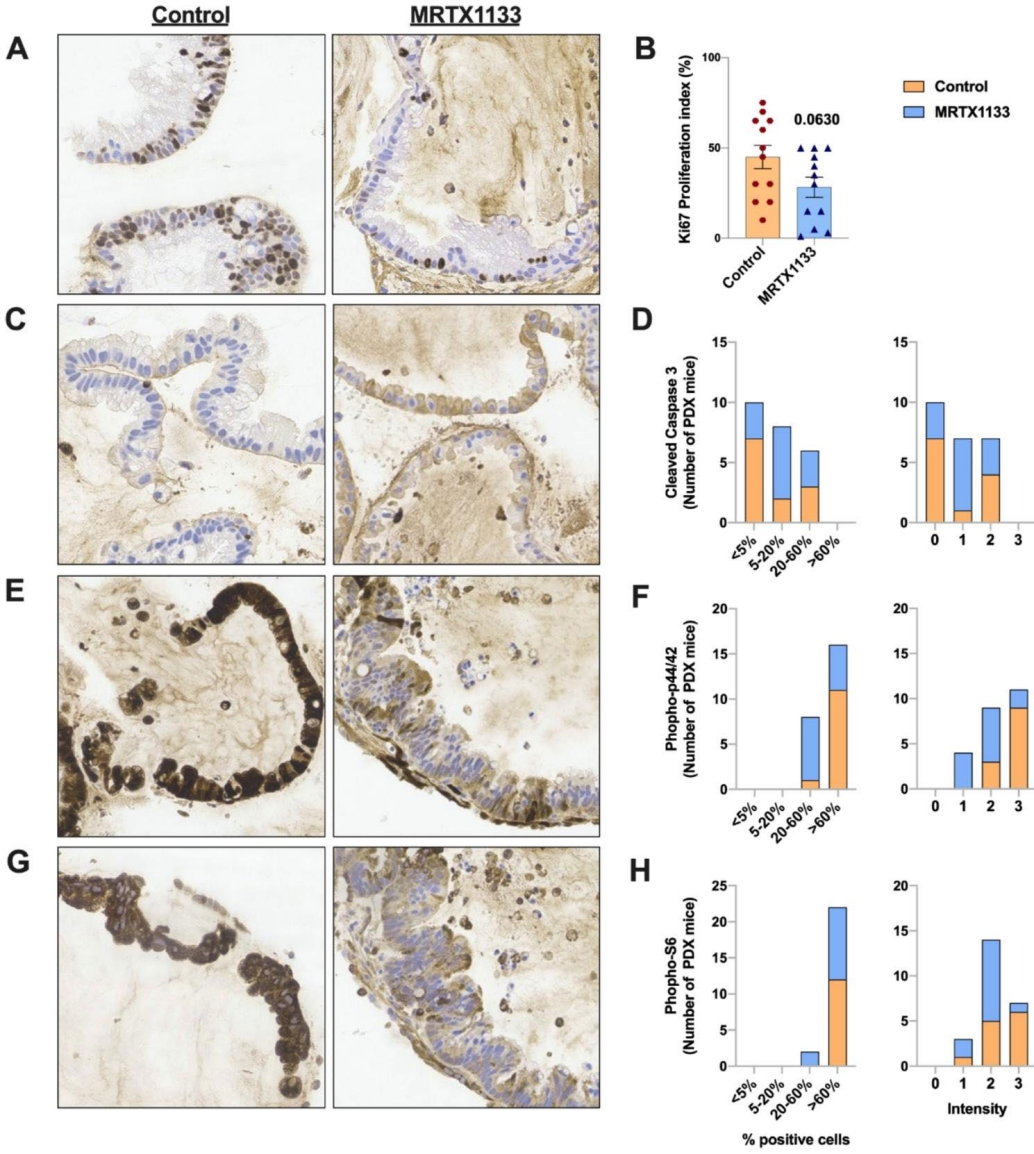
MRTX1133 reduces cell proliferation and increases apoptosis mainly through the MAPK and PI3K/AKT/mTOR signaling pathways. **(A)** Representative 20X immunohistochemical images of tumor sections stained for Ki67 from MRTX1133-treated and control mice. **(B)** Quantification of the Ki67 proliferation index in treated (n = 12) and control mice (n = 12). **(C, E, G)** Representative 20X immunohistochemical images of tumor sections stained for cleaved caspase-3, phospho-p44/42 (pERK1/2) and phospho-S6 from MRTX1133-treated and control mice. **(D, F, H)** Quantification of the percentage of positive cells and intensity of staining for cleaved caspase-3, phospho-p44/42 (pERK1/2) and phospho-S6 from MRTX1133-treated (n = 12) and control mice (n = 12). Data are represented as the mean ± SEM or stacked bar graphs (orange bars are control and blue bars are treated mice).

To better understand how MRTX1133 reduces tumor growth, we explored the Ki67 proliferation index and cleaved caspase-3 protein levels as key indicators of cell proliferation and apoptosis. We observed an alteration in both protein staining levels in MRTX1133-treated HG-PMP PDX mice (Ki67 proliferation index reduction and cleaved caspase-3 increase; Fig. 2A-D), supporting the profound tumor growth inhibition observed in these mice. Consistent with these data, a reduction in the Ki67 proliferation index and an increase in cleaved caspase-3 levels have been reported in orthotopic pancreatic HPAC and AsPC-1 cell line xenograft models [7]. Similarly, a reduction in the Ki67 proliferation index was found in the pancreatic 6419c5 cell line in immunocompetent C57BL/6 mouse models, which harbor immunotherapy-resistant pancreatic tumors [9].

Mutant KRAS, specifically KRAS^G12D^, leads to increased levels of KRAS-GTP, which results in the elevation of the PI3K/AKT and ERK pathways [12]. Based on this information, we investigated the role of these pathways in inhibiting tumor growth and observed a reduction in positive cells and staining intensity for both pERK1/2 and p-S6, with pERK1/2 being the most reduced (Fig. 2E-H). These results are in line with the reduction in pERK1/2 and p-S6 observed in vitro in both human and murine KRAS^G12D^-mutant cell lines and in the orthotopic pancreatic HPAC xenograft mouse model [7, 9].

Collectively, we present novel and original information on a striking and consistent reduction in mucinous tumor growth associated with a reduction in KRAS-dependent signaling and induction of apoptosis in a KRAS^G12D^-mutated HG-PMP xenograft mouse model using the MRTX1133 inhibitor. These results could pave the way to test this promising therapeutic option in a phase I/II clinical trial in KRAS^G12D^-mutated PMP patients to prevent relapse after surgery, as well as to test its potential effects in other more prevalent mucinous carcinomatosis, such as KRAS^G12D^-mutated mucinous colorectal cancer.

### Electronic supplementary material

Below is the link to the electronic supplementary material.

Supplementary Material 1

## Data Availability

The data generated in this study are available within the article and its supplementary data files. Nevertheless, all data are also available upon request from the corresponding author.

## Abbreviations

PMP: Pseudomyxoma peritonei
MRI: Magnetic resonance imaging
PSOGI: The Peritoneal Surface Oncology Group International
LG: Low grade
HG: High grade
CRS: Cytoreductive surgery
HIPEC: Hyperthermic intraperitoneal chemotherapy
MUC2: Mucin 2
CK7: Cytokeratin 7
CK20: Cytokeratin 20
CDX2: Homeobox protein CDX2
PDX: Patient-derived xenograft
MAPK: Mitogen-activated protein kinases
GTP: Guanosine triphosphate
