# Supplementary material for: Antitumor effect of a small-molecule inhibitor of KRASG12D in xenograft models of mucinous appendicular neoplasms
Source: Exp Hematol Oncol. 2023 Dec 8;12:102. doi: 10.1186/s40164-023-00465-4 (PMC10704766; doi:10.1186/s40164-023-00465-4)
Supplement: Supplementary file 1 — Supplementary Material 1 [file 40164_2023_465_MOESM1_ESM.docx]

**SUPLEMENTARY MATERIAL AND METHODS**

***Animals***

Nude (Foxn1nu) BALB/c mice were bred and maintained in a specific pathogen-free barrier facility at the Maimonides Institute of Biomedical Research of Cordoba (IMIBIC), and food and water were provided *ad libitum*. For PMP xenograft model generation, 6–9-week-old mice were used. Briefly, fresh tumor tissue obtained from PMP patients treated at the Reina Sofía University Hospital’s Surgical Oncologic Unit was cut into 3 × 3 × 3 mm pieces and implanted intraperitoneally in nude mice following the procedure of Flatmark et al*.* [1]. Currently, we have established an HG-PMP xenograft mouse model in our laboratory, which is characterized by <50% signet ring cells. This mouse model was maintained by mucin transplantation from one nude mouse to another. After a few passages, mucinous ascites dominated the growth pattern, and tissue was transferred to the next mouse by injecting 250 µL of mucin directly into the peritoneal cavity. The well-being of the mice was carefully monitored, and the animals were sacrificed when signs of disease were detected. Increased abdominal volume proved to be the most reliable indicator of tumor growth, and if no other symptoms were detected, the animals were sacrificed when a distinct increase in abdominal size was detectable, typically 3 weeks following implantation.

All animal experiments and procedures were approved by the Ethics Committee for Animal Experimentation at the University of Cordoba. Furthermore, permission was obtained from the Ministry of Agriculture to develop preclinical models (certificate number 12/12/2019/196).

***HG-PMP xenograft mouse model validation***

Mucinous tumor tissues from the patient’s original tumor and tumor-bearing mice were fixed in 10% neutral buffered formalin, transferred to 70% ethanol, and processed for paraffin embedding. Tissues were sectioned at 5 µm and stained with hematoxylin and eosin (H&E). In addition, the sections were immunostained with monoclonal antibodies against MUC2, CK7, CK20, P53, and CDX2 (all used at 1:100). Positive controls were included for all antibodies, with satisfactory results. Immunohistochemistry was performed on sections from the block with the most cellular tumor tissue, and the entire section was evaluated to establish an immunoreactive score. All sections were reviewed by two board-certified pathologists. The number of immunoreactive tumor cells was scored as a positivity index for P53 by referring to the number of positive cells to the total number of cells. In addition, MUC2, CK7, CK20, and CDX2 were scored as positive (weak or strong) versus negative staining.

***DNA extraction and amplicon-based next-generation sequencing (NGS)***

DNA extraction from the HG-PMP xenograft mouse model was performed using the QIAamp DNA Mini kit (Qiagen), according to the manufacturer’s instructions, but incubated with proteinase K overnight. The samples were quantified using a NanoDrop 2000 spectrophotometer (Thermo Fisher Scientific, Waltham, MA, US) and sent to the Sequencing Multiplex Company (Valencia, Spain) for library construction, sequencing, and analysis. Amplicon libraries were built for p. G12 and p. G13 *KRAS* and for p. R201 *GNAS-*specific positions (length 2 × 150 bp). Sequencing was performed using the MiSeq System (Illumina Platform). The human genome GRCh38 was used as a reference. Alignment to GRCh38 and somatic variant calling were performed. Variant calls were validated by visual inspection using Integrative Genomics Viewer. Table S1 presents the results.

***In vivo treatment***

Seven- to nine-week-old nude mice were injected with 250 µL of mucin from the previously validated HG-PMP xenograft mouse directly into the peritoneal cavity. At the same time, the mice were i.p. injected with either vehicle [10% research-grade Captisol; MedChem Express, New Jersey, US) in 50 mM citrate buffer (pH 5.0; Sigma Aldrich, Missouri, US)] or vehicle in combination with MRTX1133 (TargetMol, Massachusetts, US) at 30 mg/kg. Consistent with previous data [2,3], MRTX1133 was well tolerated with no signs of toxicity. In general, all mice were closely monitored and sacrificed by cervical dislocation after 19.25 ± 1.18 days when a distinct increase in abdominal size was detectable in the vehicle-treated group. For the experiments, a similar number of females and males were used. After sacrifice, the abdominal girth was measured, and an MRI scan was performed. Additionally, all mucinous tumors were collected and weighed. A portion of the mucinous tumor was fixed for histological analysis, and the rest was frozen at -80°C.

MRI images were acquired using a preclinical PET/MRI system (Bruker BioSpin MRI GmbH, Ettlingen, Germany, software ParaVision 3.3). Before MRI, the mice were sacrificed and placed in a supine position in a 40 mm diameter coil into the equipment. A localizer sequence was used to confirm the appropriate position of the mouse. Abdominal images were obtained with a T2_RARETURBO sequence [repetition time (TR) = 3138.902 ms, echo time (TEeff) = 48 ms, and number of averages (NA) = 8] in a coronal orientation with a field of view (FOD) of 40-45 x 40 mm, a matrix size of 128 × 128, and a slice thickness of 0.7 mm. After MRI, images were used to design a 3D model of the mouse peritoneal cavity to quantify the tumor volume using 3Dslicer v.5.2.2.

***IHC staining and analysis***

Mucinous tumor tissues were fixed in 10% neutral buffered formalin, transferred to 70% ethanol, and processed for paraffin embedding. Tissues were sectioned at 5 µm and stained with hematoxylin and eosin (H&E). In addition, the sections were immunostained with monoclonal antibodies against phospho-ERK1/2 (4370S; Cell Signaling; 1:100), phospho-S6 (2211S; Cell Signaling; 1:100), cleaved caspase-3 (9661S; Cell Signaling; 1:100), and Ki67 (Leica; 1:100). Immunohistochemistry was performed on the block with the most cellular tumor tissue, and the entire section was evaluated to establish an immunoreactive score. Manual pathology scoring was performed by two board-certified pathologists who were blinded to the treatment conditions. The number of immunoreactive tumor cells was scored as a proliferative index as a percentage of Ki67. For the remaining markers, samples were scored as the percentage of positive cells (0 = < 5% positive cells; 1 = 5-20% positive cells; 2 = 20-60% positive cells; 3 = >60% positive cells) or intensity of the staining (0 = negative; 1 = weak; 2 = moderate; 3 = strong).

***Statistical analysis***

All statistical analyses were performed using Prism v.9.3.1 software (GraphPad Software, La Jolla, CA, USA). All data are presented as the mean ± standard error of the mean (SEM). Unpaired parametric *t* test or nonparametric Mann‒Whitney *U* test was performed according to normality, assessed by the Kolmogorov‒Smirnov test. Statistical significance was set at p < 0.05.

***References***

1. Flatmark K, Reed W, Halvorsen T, Sørensen O, Wiig JN, Larsen SG, et al. Pseudomyxoma peritonei – two novel orthotopic mouse models portray the PMCA-I histopathologic subtype. BMC Cancer [Internet]. 2007;7:116–116.

2. Hallin J, Bowcut V, Calinisan A, Briere DM, Hargis L, Engstrom LD, et al. Anti-tumor efficacy of a potent and selective non-covalent KRASG12D inhibitor. Nat Med. 2022;28:2171–82.

3. Kemp SB, Cheng N, Markosyan N, Sor R, Kim I-K, Hallin J, et al. Efficacy of a small molecule inhibitor of KrasG12D in immunocompetent models of pancreatic cancer. Cancer Discov. 2022;13:298–311.

**Table S1**: Validation of the human HG-PMP xenograft mouse model.

| *Sample* | *HE* | *MUC2* | *CK7* | *CK20* | *P53 (%)* | *CDX2* |
| --- | --- | --- | --- | --- | --- | --- |
| Human PMP tissue | HG (<50% SRC) | SP | N | N | 80 | SP |
| PDX mouse 1 | HG (<50% SRC) | SP | N | N | 65 | WP |
| PDX mouse 2 | HG (<50% SRC) | SP | N | N | 60 | WP |
| PDX mouse 3 | HG (<50% SRC) | SP | N | N | 80 | WP |
| PDX mouse 4 | HG (<50% SRC) | SP | WP | WP | 60 | N |
| PDX mouse 5 | HG (<50% SRC) | SP | N | N | 85 | SP |
| PDX mouse 6 | HG (<50% SRC) | SP | N | N | 90 | SP |
| PDX mouse 7 | HG (<50% SRC) | SP | N | N | 90 | SP |
| PDX mouse 8 | HG (<50% SRC) | SP | N | N | 80 | SP |
| PDX mouse 9 | HG (<50% SRC) | SP | WP | WP | 90 | SP |
| PDX mouse 10 | HG (<50% SRC) | SP | N | N | 90 | SP |
| PDX mouse 11 | HG (<50% SRC) | SP | N | N | 90 | SP |
| PDX mouse 12 | HG (<50% SRC) | SP | N | N | 85 | SP |

N: negative; WP: weak positive; SP: strong positive.

**Table S2:** Details of genomic alterations detected with NGS.

| Gene | Accession | Mutation | Coordinate | Tumor type | Call | Coverage | Frequency (%) |
| --- | --- | --- | --- | --- | --- | --- | --- |
| KRAS | NP_004976.2 | p.G12D | 12:2245350 | HG-PMP | C/T | 6834 | 99.72 |
| GNAS | NP_000507.1 | - | 20:58909366 | HG-PMP | - | 5516 | 0 |

**Figure S1**

**Figure S1. Validation of the HG-PMP xenograft mouse model.** Representative 20X images of immunohistochemical staining for MUC2, CK7, CK20, P53 and CDX2 and hematoxylin-eosin staining in both human and PDX mouse tumor tissues.

**Figure S2**

**
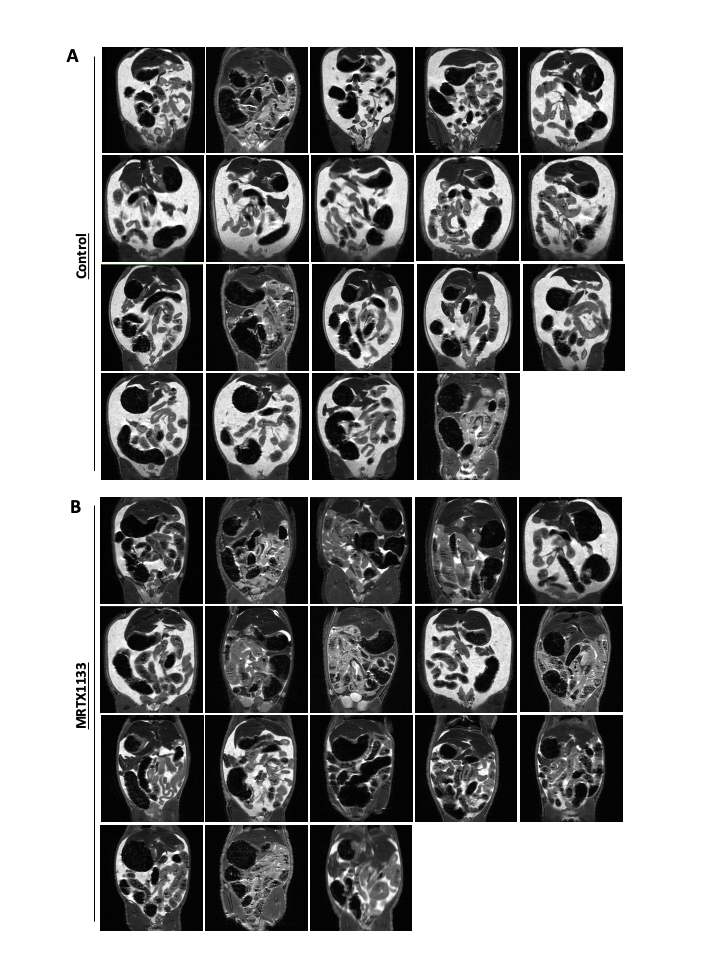
**

**Figure S2:** Representative MRI T2-weighted images of control **(A)** and MRTX1133-treated **(B)** mice. Mucin appears as hypointense regions in the images.
